# Underestimation of Leptospirosis Incidence in the French West Indies

**DOI:** 10.1371/journal.pntd.0004668

**Published:** 2016-04-29

**Authors:** Sylvie Cassadou, Jacques Rosine, Claude Flamand, Martina Escher, Martine Ledrans, Pascale Bourhy, Mathieu Picardeau, Philippe Quénel

**Affiliations:** 1 Interregional Epidemiology Unit for Antilles-Guyane, French Institute for Public Health Surveillance, Paris, France; 2 Institut Pasteur, "Biology of Spirochetes" unit, National Reference Center and WHO Collaborating Center for leptospirosis, Paris, France; 3 French Institute for Public Health Surveillance, Fort de France, France; University of California, Davis, UNITED STATES

## Abstract

**Background:**

Leptospirosis is a neglected zoonosis affecting mainly tropical and subtropical regions worldwide, particularly South America and the Caribbean. As in many other countries, under-reporting of cases was suspected in the French West Indies because of inadequate access to diagnostic tests for the general population.

**Methodology/Principal findings:**

In order to estimate the real incidence of leptospirosis in Guadeloupe and Martinique, a study was performed in 2011 using the three prevailing available biological tests for diagnosis: Microscopic Agglutination Test (MAT), IgM ELISA and PCR. The study investigated inpatients and outpatients and used active case ascertainment from data provided by a general practitioners’ sentinel network. The epidemiology of the disease was also described in terms of severity and demographic characteristics. Leptospirosis incidence was estimated at 69.4 (95%CI 47.6–91.1) and 60.6 (95%CI 36.3–85.0) annual cases per 100 000 inhabitants in Guadeloupe and Martinique, respectively, which was 3 and 4 times higher than previous estimations.

**Conclusion/Significance:**

Inclusion of PCR and IgM ELISA tests for diagnosis of leptospirosis resulted in improved sensitivity in comparison with MAT alone. Our results highlighted the substantial health burden of the disease in these two territories and the importance of access to appropriate laboratory tests. Based on our results, PCR and IgM ELISA tests have now been included in the list of tests reimbursed by the national system of social security insurance in France. Our results also underline the relevance of implementing an integrated strategy for the surveillance, prevention and control of leptospirosis in the French West Indies.

## Author Summary

Leptospirosis is a common disease in tropical regions around the world. It is caused by a bacteria excreted in environmental waters by mammals, especially rodents, through their urine. Leptospirosis has symptoms similar to other tropical diseases, including dengue fever, and early laboratory diagnosis is crucial to provide both appropriate treatments for patients and rapid control measures when an outbreak occurs. In 2011, we undertook a study to determine the incidence of leptospirosis in two territories (Guadeloupe and Martinique) in the French West Indies by establishing a surveillance network and implementing new diagnostic assays in order to ensure an exhaustive diagnostic analysis. We concluded that leptospirosis was previously significantly under-reported in the French West Indies and we recommended: 1- access to these new diagnostic tests for the entire population for a better detection of leptospirosis patients and, 2- the implementation of an integrated surveillance, alert and prevention system for the disease in this region. Our findings had raised the awareness of this neglected disease in the French West Indies and, as another consequence; new diagnostic tests are now reimbursed by the social security insurance in France.

## Introduction

Leptospirosis is a zoonotic bacterial disease which is particularly widespread in tropical and subtropical regions. It produces a wide array of clinical symptoms, ranging from an undifferentiated mild fever to severe multi-organ failure [1]. None of these symptoms is specific to the disease. Until recently, diagnosis was mostly based on serological tests, as antibodies are detectable in blood by the second week after the onset of symptoms. PCR-based methods are becoming more widely used for the detection of bacterial, in part because of their superior sensitivity and ability to establish an early diagnosis. The disease can usually be cured in humans within a few weeks without sequelae using appropriate antibiotic therapy [2]. However, it often requires hospitalization during the acute phase and complications related to the disease can be fatal. Leptospirosis disease burden estimates have been recently updated by Costa et al. [3]. The authors estimate that worldwide 1.03 million cases and 58,000 deaths occur annually. In addition, the social cost in years of potential life lost and hospital costs associated with leptospirosis are high when compared with the cost of early treatment and prevention of the infection [4,5]. However, leptospirosis is underdiagnosed worldwide, especially in low-resource tropical countries [6].

The bacteria *Leptospira* [1] are maintained in nature through the chronic renal infection of host animals, and are excreted in the hosts’ urine. Leptospirosis is transmitted through contact of abraded skin or mucous membranes either directly with infected urine or organs or indirectly with contaminated soil or water. Accordingly, activities which bring humans into contact with such contaminated environments increase the risk of contracting the disease. These include farming, gardening, building work, animal husbandry, hunting, fishing and water sports in fresh water environments.

This study focused on the two French overseas territories of Guadeloupe and Martinique (approximately 400,000 inhabitants in each) which are located in the French West Indies (hereafter FWI). Guadeloupe is an archipelago which includes two main islands, Grande-Terre and Basse-Terre (hereafter jointly covered by the name “Guadeloupe”). Guadeloupe and Martinique have a similar tropical climate with a rainy season between July and December. Furthermore, the populations are quantitatively and qualitatively similar as far as ethnic and socio-economical characteristics are concerned The population of the FWI is mainly of African or mixed descent. There are also Europeans, Indians, Lebanese, Syrians, Chinese, and Amerindians (remnants of the original pre-European population). Life expectancy at birth for males in 2013 was 76 and 79 years, respectively, in Guadeloupe and Martinique, and 85 for females in both territories. The service sector dominates the economy of the two territories while the public sector is the major employer accounting for 42% of total salaried workers. The economy is very dependent on France for subsidies and imports. In 2013, unemployment rates were 25.5% and 22%, respectively, in Guadeloupe and Martinique, 2.5 times higher than in mainland France.

Because of the warm climate in the FWI, outdoor activities are common throughout the year and are easier to undertake without the use of protection (boots, gloves, etc.). The tropical climate promotes the survival of leptospires and their proliferation in wet environments. In addition, numerous Caribbean mammals are hosts to pathogenic *Leptospira* species including rodents (most frequently), opossums, mongoose, bats, pigs, bovines, goats and dogs [7]. The *Leptospira* genus includes ten pathogenic species [8]. The most frequent serogroups in Guadeloupe and Martinique are Icterohaemorrhagiae, Canicola and Sejroe. The serogroup Ballum is also frequently reported in Guadeloupe [9].

Between 2002 and 2008, the estimated annual incidence of leptospirosis in Guadeloupe (22.5 per 100,000 inhabitants) and Martinique (13.9 per 100,000 inhabitants) was much higher than that observed in mainland France (0.47 per 100,000 inhabitants) [10]. At that time, biological diagnosis in Guadeloupe and Martinique was performed by sending blood samples to the National Reference Center for leptospirosis in Paris (Pasteur Institute) for microscopic agglutination test (MAT). Because of these logistical and technical limitations, as well as a lack of epidemiological surveillance, it was expected that the disease was underdiagnosed and its public health burden underestimated in the FWI.

In this context, an incidence study was performed in 2011: i) to obtain reliable data and assess the “real” disease burden of leptospirosis in the FWI; ii) to provide scientific evidence for the hypothesis that leptospirosis diagnosis should be reinforced by including tests capable of reliably confirming diagnosis immediately after the onset of the disease in the NABM (Nomenclature des actes de biologie médicale, which is the list of clinical pathology tests which social security insurance in France covers) and iii) to contribute to the implementation of an integrated management strategy based on an epidemiological surveillance, warning and response system.

## Methods

### Study population

The study covered the entire population of Continental Guadeloupe and Martinique. Between January 1, 2011 and December 31, 2011 (i.e., the study period), the number of incident cases of leptospirosis was counted in both territories using two sources: i) public hospitals (2 in Guadeloupe and 3 in Martinique) and ii) General Practitioners’ (GPs) sentinel surveillance networks (one on each island). In these two networks, GPs are included on a voluntary basis, according to a sampling strategy based on geographical localization and population density. The activity (annual number of consultations) of the GPs participating to these sentinel surveillance networks represents 20.4% and 22.4% of the total activity of all GPs in Guadeloupe and Martinique, respectively.

### Diagnosis strategy and case definition

Patients were eligible for inclusion in the study if they had lived permanently for one year in Martinique or Continental Guadeloupe and had consulted either a sentinel GP or a healthcare professional in a public hospital for a suspected clinical case of leptospirosis, defined as the acute onset of fever ≥38°C which then continued for less than 14 days, without any other infectious diagnosis and with at least one of the following symptoms: headache, myalgia, arthralgia and lower back pain.

The strategy used for the diagnosis of leptospirosis depended on the time of sampling, as illustrated in the accompanying supplementary document (S1 Fig). Between the first and ninth day of illness (acute phase), a real-time PCR test [11] was locally performed. If it tested negative the IgM ELISA test was performed (also locally) [12–14] and if the latter tested positive (i.e., single titer ≥1:400), definitive confirmation was obtained using a Microscopic Agglutination Test (MAT) which included a panel of 17 antigens [15]. After the ninth day of illness (immune phase), an IgM ELISA test was performed and, if it proved positive, confirmation was then obtained with a MAT. The MAT, which was performed at the National Reference Center for Leptospirosis (Institut Pasteur, Paris, France), was considered positive when the titer was ≥ 1:400 for at least one antigen (except antigen *L biflexa* serovar Patoc) [16].

If the first blood sample tested negative for all the tests, a second blood sample was recommended two weeks after the first in order to repeat the IgM ELISA. If the latter tested positive, the MAT was repeated.

Finally, leptospirosis was confirmed if the real-time PCR *or* IgM ELISA *and* MAT tests tested positive for at least one sample.

Information on gender, date of birth, city of residence, date of the onset of symptoms, date of blood sampling, laboratory test results, and, if relevant, information on hospitalization duration and disease severity, was recorded for each patient, whether the case was confirmed or not, using a standardized form. A case was defined as severe if the person died or was admitted to an intensive care unit or underwent renal dialysis or mechanical ventilation, or when a combination of these criteria was met.

### Data analysis

**Fig 1:**
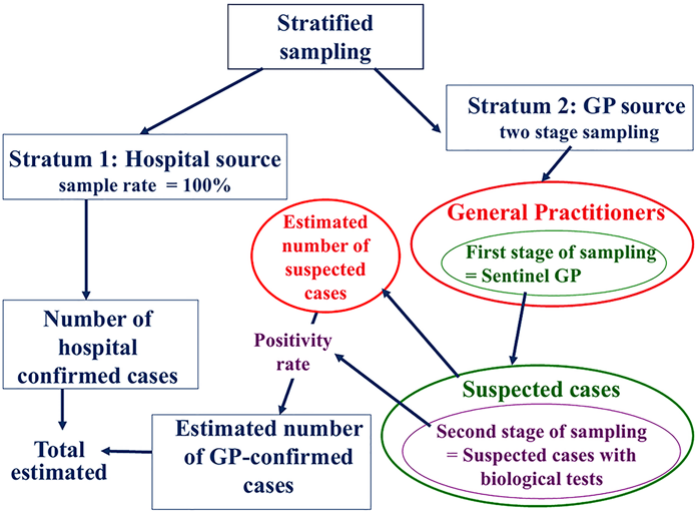
Epidemiological and statistical approach used to estimate global leptospirosis incidence, French West Indies, 2011.

The overall incidence of leptospirosis was estimated using a sampling approach, stratified on the two data sources—hospitals and GPs (Fig 1). All hospital confirmed cases (inpatients and outpatients) were taken into account for the overall incidence calculation. The number of cases confirmed outside of hospital was estimated from sentinel GPs figures, using a random two-level sampling method calculation as follows: 1) the number of cases reported by the sentinel GPs as eligible cases was extrapolated to the whole population over both territories, based on the total weekly activity rate of participating GPs (first level); 2) the number of blood samples collected by each sentinel GP for eligible cases was considered as a random sample (second level) and the positivity rate of these blood samples was applied to the previous extrapolated number.

When a patient was diagnosed by a sentinel GP and then hospitalized, he/she was considered as an hospitalized case and therefore subtracted from the GPs cases numbers.

The calculation of the confidence interval estimate took into account both the variance of the number of eligible cases reported by GPs, and the variance of the above positivity rate (S1 Protocol. Calculations for the estimation of incidence).

Differences between positivity rates according to the source of data and to the specific territory (i.e. either Guadeloupe or Martinique) were tested for their statistical significance with χ^2^ test. P values < 0.05 were considered significant.

Statistical analyses were performed using Microsoft Office Excel 2003 and Intercooled Stata 08.

### Ethics statements

This study was part of national public health surveillance program of the Institute for Public Health Surveillance (Institut de Veille Sanitaire, InVS), a governmental agency reporting to the French Ministry of Health. Therefore, consultation with ethics committee was not required. Information on leptospirosis was distributed and each participant agreed verbally an informed consent to participate as a volunteer in the study and could withdraw anytime without further obligation (S1 Consent form). Diagnostic test results was provided free of charge to the participants. The study was approved by France's data protection commission (CNIL) under number DR-2011-96 (S1 Authorization). All data used in the study was anonymized.

## Results

A total of 1,305 suspected cases were included in the study in both territories, 1,167 being recruited in hospitals and 138 through the sentinel networks. The total number of hospitalized-confirmed cases was 126 in Guadeloupe and 108 in Martinique (Table 1). By extrapolating the data reported by the sentinel networks, the total estimated number of cases was 267 and 240 in Guadeloupe and Martinique, respectively (Table 2). The corresponding overall incidence (per 100 000 inhabitants) was 69.4 and 60.6 in Guadeloupe and Martinique, respectively.

**Table 1:** Number of included patients and results of leptospirosis diagnosis in Guadeloupe and Martinique, 2011.

	Guadeloupe			Martinique		
	Hospital data (hospitalizations and consultations)	GP sentinel network	Total	Hospital data (hospitalizations and consultations)	GP sentinel network	Total
**Number of included patients**	**512**	**83**	**595**	**655**	**55**	**710**
**Number of confirmed cases (positivity rate)**	**115 (22%)**	**11 (13%)**	**126 (21%)**	**101 (15%)**	**7 (13%)**	**108 (15%)**

In total, in both territories, more than a third of biological confirmations were obtained using PCR. In Guadeloupe, secondary blood sample testing—using IgM ELISA and MAT—confirmed 31% of diagnosed cases.

**Table 2:** Indicators of the health burden of leptospirosis between 2002 and 2008, and results of the 2011 incidence study in Guadeloupe and Martinique.

	Average number per year	Average annual rate/ 100,000 inhabitants
**Guadeloupe**^a^ **2002–2008**	**99.4**	**22.5**
**Martinique 2002–2008**	**54.8**	**13.9**
**Guadeloupe**^b^ **2011**	**267 [183–351]**	**69.4 [47.6–91.1]**
**Martinique 2011**	**240 [144–337]**	**60.6 [36.3–85.0]**

^a^ Whole archipelago of Guadeloupe

^b^ continental Guadeloupe

The number of included patients (i.e. suspected cases) and the number of confirmed cases were higher in hospitals than in the sentinel networks (the latter being samples of all the GPs of each territory). Positivity rates for biological diagnosis ranged from 13 to 22% according to the data source and territory. No difference was observed between positivity rates of sentinel GPs’ patients from Guadeloupe and Martinique (13%), or between positivity rates of hospital patients and sentinel GPs’ patients (15 vs 13%) in Martinique (p>0.05). A moderate difference (22 vs 15%) was observed between the positivity rates of hospital patients from Guadeloupe and Martinique (p = 0.02), and between the positivity rates of hospital and sentinel GPs’ patients (22 vs 13%) in Guadeloupe (p = 0.05).

In Table 2, the estimated incidences in Guadeloupe and Martinique in 2011 are compared with the figures for the reference period 2002–2008. In our study in 2011, the overall estimated incidence of leptospirosis was three times higher for Guadeloupe and four times higher for Martinique compared with the reference period 2002–2008. In addition, a difference of 12% was observed between Guadeloupe and Martinique in 2011, compared with almost 40% for the reference period (p < 0.01).

In both territories, the positivity rates was most commonly observed in adults aged 20–59, than in the over-60 population (Table 3). The study also showed, for the first time in Guadeloupe and Martinique, that leptospirosis also occurs in children, with cases confirmed in persons younger than 10. Men were six times more likely than women to be affected by leptospirosis in both territories, the sex ratios of confirmed cases being similar in Guadeloupe (6.4) and Martinique (6.2). This trend was observed across all age groups, being statistically significant among adults and, in Guadeloupe, among people over 60 years.

**Table 3:** Number of included and confirmed patients, according to gender and age, gender odds ratio and confidence intervals, Guadeloupe and Martinique, 2011.

		Number of included patients		Number of confirmed cases		Odds Ratio [CI]
	Age brackets	M	F	M	F	
**Guadeloupe**	**0–19**	**86**	**52**	**7**	**1**	**4.51 [0.54–37.82]**
	**20–59**	**182**	**99**	**69**	**12**	**4.43 [2.26–8.68]**
	**60 and over**	**125**	**51**	**33**	**4**	**4.21 [1.40–12.60]**
**Martinique**	**0–19**	**64**	**43**	**5**	**2**	**1.74 [0.32–9.39]**
	**20–59**	**270**	**133**	**72**	**8**	**5.68 [2.65–12.20]**
	**60 and over**	**123**	**77**	**16**	**5**	**2.15 [0.76–6.14]**

Disease severity indicators are displayed in Table 4. In both Guadeloupe and Martinique, these indicators confirm the leptospirosis disease burden. The eight deaths which occurred in Guadeloupe were directly attributed to leptospirosis by hospital specialists in infectious diseases.

**Table 4:** Leptospirosis severity indicators, Guadeloupe and Martinique, 2011.

	Hospitalization ^a^		Severe cases^c^		Death	
	Number	Incidence ^b^	Number	Incidence^b^	Number	Case fatality rate^d^ [CI]
**Guadeloupe**	**100**	**25.9**	**20**	**5.2**	**8**	**3 [2–4]**
**Martinique**	**70**	**17.7**	**13**	**3.3**	**0**	** **

^a^ Duration > 24h,

^b^ Per 100,000 inhabitants,

^c^ Death or admission to intensive care or renal replacement therapy or mechanical ventilation or a combination of these criteria,

^d^ Percentages

## Discussion

In 2011, the estimated number of confirmed cases of leptospirosis in Guadeloupe was 267, comprising 115 hospital cases and an estimated 152 GP cases. In Martinique, 240 cases were confirmed, comprising 101 hospital cases and an estimated 139 GP cases. The total estimated number of cases for each territory–approximately 250 cases per year—was close to that observed in mainland France, where the population is approximately 120 times greater [15], indicating that the burden of leptospirosis is much higher in the FWI. The incidence in FWI remained high in the last few years (2012–2014) with a similar number of laboratory-confirmed cases in both territories.

Approximately seventy and sixty cases were estimated per 100,000 inhabitants in 2011, respectively, in Guadeloupe and Martinique. These estimates were respectively three and four times higher than each territory’s average incidence during the reference period 2002–2008 (reported by the National Reference Center) (Table 2). Our study shows that a significant higher number of cases was detected when both IgM ELISA and PCR tests are used, further indicating that the lack of adequate diagnostic tests contributes to under-reporting of cases [3].

However, some limitations of our study must be considered. First, sentinel GPs are not randomly selected. However, these n sentinel GPs networks have been widely used for a decade to estimate the numbers of suspected cases of other diseases in Guadeloupe and Martinique (including dengue and, more recently, chikungunya) and the estimates obtained from these networks were coherent with those obtained from other surveillance systems, as laboratory-based data or hospital emergency department data [17].

Second, 22% of eligible patients were not included in the study because sentinel GPs did not test them for leptospirosis. The reasons for non-inclusion were diverse (GPs in holidays, misinterpretation of the case definition or inclusion criteria, etc.) and may not introduce bias. We therefore considered that eligible patients were included at random to receive a prescription for biological testing.

Leptospirosis belongs to the group of neglected tropical diseases [3] which comprises some of the most common infections in Latin America and Caribbean countries. The 7.5% rate of severe leptospirosis (ratio of the number severe cases / total estimated number of cases) observed in this study in 2011 is much higher than the 0.3% recorded for dengue during the most recent epidemic in 2010 [17–18]. The annual incidence of leptospirosis has been shown to be associated with climate and meteorological conditions. Thus, heavy rainfall results to increased survival of *Leptospira* in the environment and increased exposure of humans to water. In 2011, both territories experienced heavy rainfalls, but no cyclone [19,20]. A long-term surveillance system would therefore be required to accurately describe annual variations in the disease incidence, for example during the El Niño Southern Oscillation periods [21].

The incidence of severe cases of leptospirosis was 5.2 in Continental Guadeloupe and 3.3 in Martinique per 100,000 inhabitants in 2011. This reported incidence is similar to the one recorded in Réunion Island in 2011where the incidence of cases transferred to intensive care (only) was 2 per 100,000 inhabitants [22].

Case fatality rates (CFR) observed in Guadeloupe (3%) and in Martinique (0%) were also similar to that observed in Réunion Island for the period 2004–2008, where it ranged from 0% to 7% depending on the year, except in 2006 when leptospirosis lethality reached 38% during the chikungunya epidemic [23]. Possibly due to the relatively higher income status of FWI and to access to early and free testing during our study period, estimated case fatality rates in FWI are lower than the global estimations of Costa et al. [3].

The demographic characteristics of the cases in our study match those described in the literature, albeit with an increased proportion of older persons in the FWI [24, 25]. The predominance of male cases is generally attributed to the hypothesis that men are potentially more exposed than women due to more frequent at-risk activities. However, we also observed this ratio in the older age group where women are more numerous than men, and probably have similar daily activities as the latter, suggesting that other hypothesis needs to be evaluated.

Improving patient care is a priority. Access to diagnosis is crucial, because treatment for leptospirosis patients is much more effective if antibiotics are administered as early as possible following the onset of disease [5,26]. Early diagnosis of acute leptospirosis by real-time PCR would prevent potential complications and limit periods of stays in hospital. This assay, which was not available in Guadeloupe before the study, is now routinely used in both territories. During the immune phase of the disease (from the end of the first week), the IgM ELISA, which usually becomes positive earlier than MAT in the course of the illness, can also offer useful support to physicians to make good treatment decisions. The IgM ELISA is a simple and rapid method which is not requiring the use of sophisticated laboratory equipment or trained personnel. Partly because of the results of this study, in September 2014 the French social security insurance decided to reimbursed the cost of both PCR and ELISA for the diagnosis of leptospirosis in mainland France and French overseas territories.

Implementation of an epidemiological surveillance system including the systematic collection and analysis of data should allow the public health community to respond more quickly to a given epidemiological situation (clustered cases, seasonal outbreaks, evaluation of prevention and control measures, etc). These results advocate for an integrated surveillance, early warning and management strategy to reduce the incidence and severity of leptospirosis. The experience of dengue, which prompted the implementation of an integrated management strategy promoted by the WHO in the FWI, could serve as a model [18, 27].

## Supporting Information

S1 Fig Algorithm for the biological diagnosis strategy. (TIF)

S1 Protocol Calculations for the estimation of incidence. (DOCX)

S1 Consent form (PDF)

S1 Authorization From the France’s data protection commission (Commission Informatique et liberté –CNIL). (PDF)

## References

[1] Glynn K, Hartskeel R, Ko A, Meslin F. Leptospirosis In: Heymann DL, editor. Control of communicable diseases manual, 19th ed. Washington: American Public Health Association; 2008 p.351–7.

[2] Spichler A, Athanazio D, Seguro AC, Vinetz JM. Outpatient follow-up of patients hospitalized for acute leptospirosis. Int J Infect Dis. 2011 7;15(7):486–90. 10.1016/j.ijid.2011.03.020 PMC3117907 21616696

[3] CostaF, Hagan JE, Calcagno J, Kane M, Torgerson P, Martinez-Silveira MS, et al (2015). Global Moàrbidity and Mortality of Leptospirosis: A Systematic Review. PloS Negl Trop Dis 9(9): e0003898 10.1371/journal.pntd.0003898 26379143 PMC4574773

[4] Souza VMM, Simoes-Arsky MLN, Barbosa de Castro AP, Navegantes de Araujo W. Years of potential life lost and hospitalization costs associated with leptospirosis in Brazil. Rev Saùde Pùblica 2011;45(6):1–7 21953079 10.1590/s0034-89102011005000070

[5] Suputtamongkol Y, Pongtavornpinyo W, Lubell Y, Suttinont C, Hoontrakul S, Phimda K et al Strategies for diagnosis and treatment of suspected leptospirosis: a cost-benefit analysis. PLoS Negl Trop Dis 4(2): e610 10.1371/journal.pntd.0000610 20186324 PMC2826401

[6] World Health Organization. Leptospirosis: an emerging public health problem. Weekly Epidemiological Record. 2011;86:45–50.

[7] Desvars A, Cardinale E, Michault A. Animal leptospirosis in small tropical areas. Epidemiol Infect. 2011;139:167–88. 10.1017/S0950268810002074 20875197

[8] Cerqueira GM, Picardeau M. A century of *Leptospira* strain typing. Infect Genet Evol. 2009;9:760–8. 10.1016/j.meegid.2009.06.009 19540362

[9] Bourhy P, Herrmann Storck C, Theodose R, Olive C, Nicolas M, Hochedez P, Lamaury I, Zinini F, Brémont S, Landier A, Cassadou S, Rosine J, Picardeau M. Serovar diversity of pathogenic *Leptospira* circulating in the French West Indies. PLoS Negl Trop Dis. 2013;7(3):e2114 10.1371/journal.pntd.0002114 Epub 2013 Mar 14. 23516654 PMC3597474

[10] Centre national de référence de la leptospirose. Rapports d’activité de 2002 à 2008 [Internet]. Paris: Institut Pasteur; 2012 [cited 2013 Jun 21]. Available from: http://www.pasteur.fr/fr/sante/centres-nationaux-reference/les-cnr/leptospirose/rapports-d-activite. French.

[11] Merien F, Portnoi D, Bourhy P, Charavay F, Berlioz-Artaud A et al A rapid and quantitative method for the detection of *Leptospira* species in human leptospirosis. FEMS Microbiol Lett. 2005 8 1;249(1):139–47. 16006065 10.1016/j.femsle.2005.06.011

[12] Bourhy P, Vray M, Picardeau M. Evaluation of an in-house ELISA using the intermediate species *Leptospira* fainei for diagnosis of leptospirosis.J Med Microbiol. 2013 6;62(Pt 6):822–7. 10.1099/jmm.0.054304–0 Epub 2013 Mar 14. 23493028

[13] Trombert-Paolantoni S**,** Thomas P, Hermet F, Clairet V, Litou N, Maury L. Leptospirosis screening: performance of the Serion Elisa Classic Leptospira IgM KIT. Pathol Biol (Paris). 2010 2;58(1):95–9. 10.1016/j.patbio.2009.06.008 Epub 2009 Nov 4. 19892494

[14] Desakorn V, Wuthiekanun V, Thanachartwet V, Sahassananda D, Chierakul W, Apiwattanaporn A, Day NP, Limmathurotsakul D, Peacock SJ. Accuracy of a commercial IgM ELISA for the diagnosis of human leptospirosis in Thailand. Am J Trop Med Hyg. 2012 3; 86(3):524–7. 10.4269/ajtmh.2012.11–0423 22403329 PMC3284374

[15] Picardeau M, Bourhy P. Rapport d’activité 2011 [Internet]. Paris: Institut Pasteur; 2012 [cited 2013 Jun 21]. Available from: http://www.pasteur.fr/ip/resource/filecenter/do-cument/01s-00004j-015/ra-cnr-lepto-2011.pdf French.

[16] Picardeau M. Diagnosis and epidemiology of leptospirosis. Med Mal Infect 2013 1;43(1):1–9. PubMed Central PMCID: PMC3597474 23337900 10.1016/j.medmal.2012.11.005

[17] L'Azou M, Taurel AF, Flamand C, Quénel P. Recent epidemiological trends of dengue in the French territories of the Americas (2000-2012): a systematic literature review. PLoS Negl Trop Dis. 2014 11 6;8(11):e3235 10.1371/journal.pntd.0003235 eCollection 2014 Nov 25375627 PMC4222734

[18] Quénel P, Rosine J, Cassadou S, Ardillon V, Blateau A, Mathéus S et al. [Epidemiology of dengue in the French overseas departments of America]. Bull Epidemiol Hebd. 2011 Sep 20; 33–34: 358–63. French.

[19] Météo-France. Bulletin climatique annuel Guadeloupe [Internet] 2011[cited 2013 Jun]. Available from: http://www.meteo.gp/alaune/bca/archives/bca_2011_guadeloupe.pdf. French

[20] Météo-France. Bulletin climatique annuel Martinique [Internet] 2011[cited 2013 Jun]. Available from: http://www.meteo.gp/alaune/bca/archives/bca_2011_martinique.pdf. French

[21] Hermmann-Storck CH, Postic D, Lamaury I, Perez JM (2008) Changes in epidemiology of leptospirosis in 2003–2004, a two El Niño Southern Oscillation period, Guadeloupe archipelago, French West Indies. Epidemiol Infect 136: 1407–1415. 18096102 10.1017/S0950268807000052 PMC2870739

[22] Indian Ocean Cell of French Institute for Public Health Surveillance. Surveillance de la leptospirose à la Réunion: bilan 2011. Point épidémiologique [Internet] 2012 Jan [cited 2013 Jun 26] 5. Available from: http://www.invs.sante.fr/fr/Publications-et-outils/Points-epidemiologiques/Tous-les-numeros/Ocean-Indien/2012/Surveillance-de-la-leptospirose-a-la-Reunion-en-2010.-Point-epidemiologique-au-18-janvier-2011. French.

[23] Renault P, Boidin E, D’Ortenzio E, Balleydier E, Daniel B, Filleul L. Epidemiological surveillance of leptospirosis on Reunion Island in 2004–2008: possible effect of Chikungunya infection on the case fatality rate of leptospirosis. Bull Soc Pathol Exot. 2011 5; 104(2):148–52. 10.1007/s13149-010-0114-4 . French. 21174236

[24] Katz AR, Buchholtz AE, Hinson K, Park SY, Effler PV. Leptospirosis in Hawaii, USA, 1999–2008. Emerg Infec Dis. 2011;17: 221–6. 21291592 10.3201/eid1702.101109 PMC3204774

[25] Adesyun AA, Baboolal S, Suepaul S Dookeran S, Stewart-Johnson A. Human leptospirosis in the Caribbean, 1997–2005: characteristics and serotyping of clinical samples from 14 countries. Rev Panam Salud Publica. 2011;29(5):350–7 21709940

[26] Galloway RL, Levett PN, Tumeh JW, Flowers CR. Assessing cost effectiveness of empirical and prophylactic therapy for managing leptospirosis outbreaks. Epidemiol Infect. 2009 9;137(9):1323–32. 10.1017/S0950268808001751 Epub 2009 Jan 23. 19161641

[27] San Martín JL, Brathwaite-Dick O. Integrated strategy for dengue prevention and control in the Region of the Americas. Rev Panam Salud Publica. 2007 1; 21(1):55–63. Spanish. 17439693 10.1590/s1020-49892007000100011

