# Correction: Underestimation of Leptospirosis Incidence in the French West Indies

**DOI:** 10.1371/journal.pntd.0006128

**Published:** 2017-12-06

**Authors:** Sylvie Cassadou, Jacques Rosine, Claude Flamand, Martina Escher, Martine Ledrans, Pascale Bourhy, Mathieu Picardeau, Philippe Quénel

Eight authors were not included in the author byline and inadvertently listed only in the Acknowledgements. Please see the full list of added authors as well as an updated byline below:

Jean-Baptiste Adrien should be listed as the eighth author and affiliated with Emergency Department of Hospital Center Basse-Terre, Basse-Terre, Guadeloupe, France. The contributions of this author are as follows: Substantial contribution to conception of clinical procedures and acquisition of data through patients care and filling in the study form in Guadeloupe, revising the article critically for important intellectual content, and final approval of the version to be published.

André Cabié should be listed as the ninth author and affiliated with University Hospital Center Fort de France, Fort de France, Martinique, France. The contributions of this author are as follows: Substantial contribution to conception of clinical procedures and acquisition of data through patients care and filling in the study form in Martinique, revising the article critically for important intellectual content, and final approval of the version to be published.

Stephanie Guyomard should be listed as the tenth author and affiliated with Institut Pasteur de Guadeloupe, Les Abymes, Guadeloupe, France. The contributions of this author are as follows: Substantial contribution to conception of the biological procedures and acquisition of data through blood samples analysis and interpretation of biological results in Guadeloupe, revising the article critically for important intellectual content, and final approval of the version to be published.

Cecile Herrmann-Storck should be listed as the eleventh author and affiliated with University Hospital Center Pointe à Pitre, Pointe à Pitre, Guadeloupe, France. The contributions of this author are as follows: Substantial contribution to conception of the biological procedures and acquisition of data through blood samples analysis and interpretation of biological results in Guadeloupe, revising the article critically for important intellectual content, and final approval of the version to be published.

Patrick Hochedez should be listed as the twelfth author and affiliated with University Hospital Center Fort de France, Fort de France, Martinique, France. The contributions of this author are as follows: Substantial contribution to conception of clinical procedures and acquisition of data through patients care and filling in the study form in Martinique, revising the article critically for important intellectual content, and final approval of the version to be published.

Claude Olive should be listed as the thirteenth author and affiliated with University Hospital Center Fort de France, Fort de France, Martinique, France. The contributions of this author are as follows: Substantial contribution to conception of the biological procedures and acquisition of data through blood samples analysis and interpretation of biological results in Martinique, revising the article critically for important intellectual content, and final approval of the version to be published.

Rafaelle Théodose should be listed as the fourteenth author and affiliated with University Hospital Center Fort de France, Fort de France, Martinique, France. The contributions of this author are as follows: Substantial contribution to conception of the biological procedures and acquisition of data through blood samples analysis and interpretation of biological results in Martinique, revising the article critically for important intellectual content, and final approval of the version to be published.

Isabelle Lamaury should be listed as the fifteenth author and affiliated with University Hospital Center Pointe à Pitre, Pointe à Pitre, Guadeloupe, France. The contributions of this author are as follows: Substantial contribution to conception of clinical procedures and acquisition of data through patients care and filling in the study form in Guadeloupe, revising the article critically for important intellectual content, and final approval of the version to be published.

Additionally, Philippe Quénel should now be listed as the sixteenth author.

Sylvie Cassadou1*, Jacques Rosine1, Claude Flamand1¤, Martina Escher1, Martine Ledrans1, Pascale Bourhy2, Mathieu Picardeau2, Jean-Baptiste Adrien3, André Cabié4, Stéphanie Guyomard5, Cécile Herrmann-Storck6, Patrick Hochedez4, Claude Olive7, Raphaelle Théodose7, Isabelle Lamaury8, Philippe Quénel1

1 Interregional Epidemiology Unit for Antilles-Guyane, French Institute for Public Health Surveillance, Paris, France.

2 Institut Pasteur, "Biology of Spirochetes" unit, National Reference Center and WHO Collaborating Center for leptospirosis, Paris, France.

3 Emergency Department, Hospital Center Basse-Terre, Basse-Terre, Guadeloupe, France.

4 Infectious Diseases Department, University Hospital Center Fort de France, Fort de France, Martinique, France.

5 Institut Pasteur de Guadeloupe, Les Abymes, Guadeloupe, France.

6 Microbiology Laboratory, University Hopital Center Pointe à Pitre, Pointe à Pitre, Guadeloupe, France.

7 Microbiology Laboratory, University Hopital Center Fort de France, Fort de France, Martinique, France.

8 Infectious Diseases Department, University Hospital Center Pointe à Pitre, Pointe à Pitre, Guadeloupe, France.

¤a Current address: Epidemiology Unit of Pasteur Institute of French Guiana, Cayenne, France ¤b Current address: Ecole des Hautes Etudes en Santé Publique, Laboratoire d'étude et de recherche en environnement et santé, Rennes, France.
