# Supplementary figures and images for: Underestimation of Leptospirosis Incidence in the French West Indies
Source: PLoS Negl Trop Dis. 2016 Apr 29;10(4):e0004668. doi: 10.1371/journal.pntd.0004668 (PMC4851364; doi:10.1371/journal.pntd.0004668)

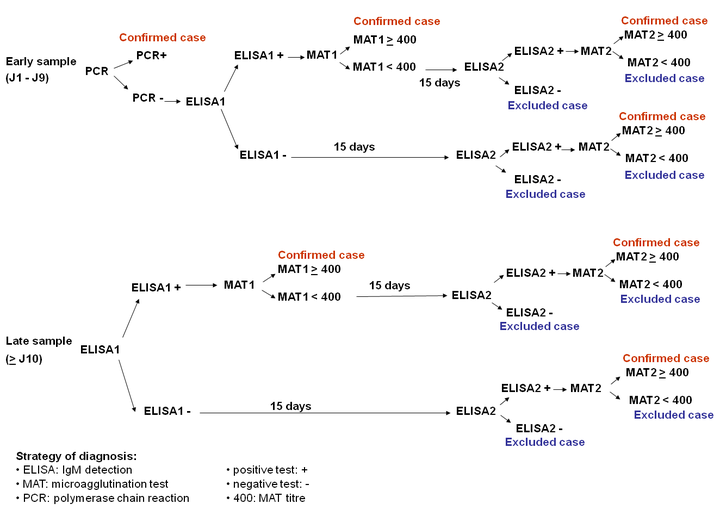

Supplement: S1 Fig — (TIF) [file pntd.0004668.s001.tif]
