# Supplementary material for: Underestimation of Leptospirosis Incidence in the French West Indies
Source: PLoS Negl Trop Dis. 2016 Apr 29;10(4):e0004668. doi: 10.1371/journal.pntd.0004668 (PMC4851364; doi:10.1371/journal.pntd.0004668)
Supplement: S1 Protocol — (DOCX) [file pntd.0004668.s002.docx]

**S 1– Protocol for the estimation of the incidence**

1. **Weekly estimation of the number of cases**
   1. **Central estimation**

From the sentinels GPs source, the weekly number of suspected cases is extrapolated to all the GPs of the territory as following:

$$Ns=\sum_{i=1}^{k_{d}} Xi\frac{A}{\sum_{i=1}^{k_{d}} a_{i}}$$

$Ns$ Extrapolated number of suspected cases, week *S*, to all the GPs of the territory

*$k_{d}$*  Number of sentinels GPs responding for the week *S*

*$Xi$* Number of suspected cases reported by the sentinel GP*i* for the week *S*

** Total annually number of consultations for GP*i*, responding for the week *S*

*$A$* Total annually number of consultations for all the GP’s of the territory

Based on the results of the biological diagnosis performed on the samples taken off during the week *S*, and prescribed by the sentinel GPs, the positivity rate of the week S is calculated and then applied to the total number of suspected cases estimated for the week *S* ($Ns$).

$$Nlmgs=Txps\times Ns$$

$Nlmgs$ Number of confirmed cases, estimated for all the GPs of the territory, for the week *S*

$Txps$Positivity rate of the biological diagnosis prescribed by sentinel GPs, during the week S

$Ns$ Extrapolated number of suspected cases, week *S*, to all the GPs of the territory

Then, this estimated number of confirmed cases is added to the number of cases confirmed in hospital (inpatients and out patients) during the week *S*.

$$Nls=Nlmgs+Nlhs$$

$Nls$Number of confirmed cases on all the territory during the week S

$Nlmgs$ Number of confirmed cases, estimated for all the GPs of the territory, for the week *S*

$Nlhs$ Number of confirmed cases reported by hospitals, for the week *S*

- 1. **Confidence interval calculation:**

The calculation of the confidence interval took firstly into account the variance in the number of suspected clinical cases linked to the statistical sampling of sentinel GPs *$V(Ns)$*, and secondly the variance in the positivity rate of the biological diagnosis linked to the sampling of patients really taken off among the eligible patients of the sentinel GPs *$V(Txp)$*.

*$V(Ns\times Txps)=\left(Ns\times Txps\right)^{2}\times\left(\frac{V(Ns)}{Ns^{2}}+\frac{V(Txps)}{Txps^{2}}\right)$*

*$V(Ns\times Txps)$* Global variance of the number of confirmed cases estimated for the week *S*.

$Ns$ Extrapolated number of suspected cases, week *S*, to all the GPs of the territory

$Txps$ Positivity rate of the biological diagnosis prescribed by sentinel GPs, during the week S

*$V(Ns)$* Variance of the Extrapolated number of suspected cases, week *S*, to all the GPs of the territory

**Variance of the positivity rate of the biological diagnosis prescribed by sentinel GPs, during the week S

Finally, the limits of the confidence interval, for each weekly estimation, are calculated as following:

*$IC=Nls\pm1,96\sqrt{V(Ns\times Txps)}$*

$Nls$ Number of confirmed cases on all the territory during the week S

*$V(Ns\times Txps)$* Global variance of the number of confirmed cases estimated for the week *S*.

1. **Calculation of the cumulated estimation**

The estimations of the cumulated number of confirmed cases, occurred during a P period, are calculated using the same formulas:

1. The extrapolation of the all suspected cases reported by sentinel GPs during the period P to all the GPs of the territory, during the same period, based on the ratio of the numbers of consultations ;
2. The global positivity rate of all the samples tested during the period P ;
3. The addition of this estimated number and the number reported by hospitals.
