# Supplementary material for: Underestimation of Leptospirosis Incidence in the French West Indies
Source: PLoS Negl Trop Dis. 2016 Apr 29;10(4):e0004668. doi: 10.1371/journal.pntd.0004668 (PMC4851364; doi:10.1371/journal.pntd.0004668)
Supplement: S1 Consent form — (PDF) [file pntd.0004668.s003.pdf]

ARS Agence de Santé Guadeloupe Saint-Martin Saint-Barthélemy

INSTITUT DE VEILLE SANITAIRE
CIRE ANTILLES-GUYANE

Information sur le signalement des cas de LEPTOSPIROSE en Guadeloupe

---

Votre médecin vous a prescrit ou a prescrit à votre enfant une prise de sang pour le diagnostic de la leptospirose.

Qu’est ce que la leptospirose ?
La leptospirose est une maladie infectieuse, due à une bactérie (*Leptospira interrogans*). Les animaux en particulier les rongeurs (rats) constituent le principal réservoir bactérien. Ces animaux rejettent les bactéries Leptospira dans le milieu extérieur par leurs urines. L'homme se contamine le plus souvent par contact cutané ou muqueux (au niveau d'une plaie ou par immersion longue) avec une eau ou de la boue infectée par la bactérie.

A qui sont signalés les cas de leptospirose ?
Les signalements sont effectués par le laboratoire d’analyses au Médecin Inspecteur de Santé Publique de l’ARS (Agence Régionale de la Santé).

Quelles informations sont transmises ?
En cas de diagnostic positif, le laboratoire notifie le résultat au médecin de l’ARS, le nom du patient et son adresse ; ces données sont transmises au service de veille sanitaire de l’ARS.

A quoi sert le signalement ?
Le signalement sert à mettre en place une intervention du service de veille et de gestion sanitaire de l’ARS au domicile du patient et dans sa périphérie proche (quartier) afin de rechercher des sources éventuelles d’exposition. Ces actions servent à prévenir la transmission de la maladie chez d’autres personnes de l’entourage familial ou dans le quartier. Ce service est entièrement gratuit.

Avant toute intervention, un agent vous contactera pour convenir d’un rendez-vous.

Conformément aux articles 39 et suivants de la loi n° 78-17 du 6 janvier 1978 relative à l’informatique, aux fichiers et aux libertés, toute personne peut obtenir communication et, le cas échéant, rectification ou suppression des informations la concernant, en s’adressant par courrier à la Cire Antilles-Guyane ou à la Cellule de Veille Sanitaire de l’ARS. Toute personne peut également, pour des motifs légitimes, s’opposer au traitement des données la concernant.

Pour toute information complémentaire, vous pouvez vous adresser

- **à votre médecin traitant**
- **à l’ARS de Guadeloupe**

Cellule de veille sanitaire - Téléphone : 05 90 99 49 23/25
Rue des Archives
97 113 Gourbeyre
