# Supplementary material for: Underestimation of Leptospirosis Incidence in the French West Indies
Source: PLoS Negl Trop Dis. 2016 Apr 29;10(4):e0004668. doi: 10.1371/journal.pntd.0004668 (PMC4851364; doi:10.1371/journal.pntd.0004668)
Supplement: S1 Authorization — (PDF) [file pntd.0004668.s004.pdf]

CNIL

Le Vice-Président délégué

Madame Françoise WEBER
INSTITUT DE VEILLE SANITAIRE
INVS
SFLE - SERVICE FINANCIER LOGISTIQUE ET ECONOMIQUE
12 RUE DU VAL D'OSNE
94415 - SAINT MAURICE CEDEX

Paris. le 18 MARS 2011

N/Réf. : EGY/FLR/AR112475

Objet : NOTIFICATION D'AUTORISATION

Décision DR-2011-096 autorisant l'INSTITUT DE VEILLE SANITAIRE à mettre en œuvre un traitement de données ayant pour finalité une étude d'incidence de la leptospirose aux antilles afin d'estimer de façon fiable l'impact de cette maladie et d'articuler de la façon la plus réactive possible la détection des cas et les actions environnementales ciblées (Demande d'autorisation n° 1438809)

Madame La Directrice,

Vous avez saisi notre Commission d'une demande d'autorisation relative à un traitement de données à caractère personnel ayant pour finalité :

ÉTUDE D'INCIDENCE DE LA LEPTOSPIROSE AUX ANTILLES

Ce traitement relève de la procédure des articles 54 et suivants de la loi du 6 janvier 1978 modifiée.

Les services de notre Commission ont étudié les conditions définies par le dossier de formalités préalables déposé à l'appui de cette demande et notamment celles relatives à l'exercice effectif des droits des participants à l'étude.

Après avoir examiné les catégories de données traitées et les destinataires, je vous rappelle que conformément au 3ème alinéa de l'article 55, la présentation des résultats du traitement de données ne peut, en aucun cas, permettre l'identification directe ou indirecte les personnes concernées.

En application des articles 15 et 69 de la loi précitée et de la délibération n° 2009-674 du 26 novembre 2009 portant délégation d'attributions de la Commission nationale de l'informatique et des libertés à son président et à son vice-président délégué, j'autorise la mise en œuvre de ce traitement.

Je vous prie, Madame, d'agréer l'expression de mes salutations distinguées.

E. de Givry

Emmanuel de GIVRY
